# FEM Analysis of Dental Implant-Abutment Interface Overdenture Components and Parametric Evaluation of Equator^®^ and Locator^®^ Prosthodontics Attachments

**DOI:** 10.3390/ma12040592

**Published:** 2019-02-16

**Authors:** Marco Cicciù, Gabriele Cervino, Dario Milone, Giacomo Risitano

**Affiliations:** 1 Department of Biomedical and Dental Sciences and Morphological and Functional Imaging, Messina University, 98100 Messina, Italy; gcervino@unime.it; 2 Department of Engineering, Messina University, 98100 Messina, ME, Italy; dario.milone94@gmail.com (D.M.); grisitano@unime.it (G.R.)

**Keywords:** overdenture attachments, implant abutment connections, stress distribution, FEM

## Abstract

The objective of this investigation was to analyze the mechanical features of two different prosthetic retention devices. By applying engineering tools like the finite element method (FEM) and Von Mises analyses, we investigated how dental implant devices hold out against masticatory strength during chewing cycles. Two common dental implant overdenture retention systems were analyzed and then compared with a universal—common dental abutment. The Equator^®^ attachment system and the Locator^®^ arrangement were processed using the FEM Ansys^®^ Workbench. The elastic features of the materials used in the study were taken from recent literature. Results revealed different responses for both the devices, and both systems guaranteed a perfect fit over the axial load. However, the different design and shape involves the customized use of each device for a typical clinical condition of applying overdenture systems over dental implants. The data from this virtual model showed different features and mechanical behaviors of the overdenture prosthodontics attachments. A three-dimensional system involved the fixture, abutment, and passant screws of three different dental implants that were created and analyzed. Clinicians should find the best prosthetic balance to better distribute the stress over the component, and to guarantee the patients clinical long-term results.

## 1. Introduction

The management of the atrophic mandible using dental implants is a common technique. The lower jaw is a complex anatomical district and the presence of the tongue reduces the contact surface of the removable prosthesis [1,2]. Recently, the possibility of positioning two or more dental implants in the anterior mandible gives clinicians the opportunity to increase the removable prosthesis retention or to fix partial or complete lower dentures [1,2,3,4,5]. Quality assurance of health care delivery has emphasized the importance of the patient’s perceptions of medical therapies since the early seventies. The patient’s desires, both before and after a medical therapy, are fundamental to the final satisfaction with the treatment outcomes [6,7,8,9]. This is even more critical today, as the current practice of evidence based medicine requires that patients be actively engaged in the decision making process with regards to their treatment. Moreover, evaluating the expectations of patients before the treatment starts appears to be an essential prerequisite to achieve successful patient reports on long-term clinical outcomes [10,11]. Though removable prosthesis can offer high aesthetics, the main limit of such dental rehabilitation is related to their retention. Dental implants and related prosthodontics treatment offer high levels of oral health linked to the quality of life, and are particularly important in times of population aging as the edentulousness percentage continues to be relevantly high [12,13,14,15]. The best attachment system choice is also related to the duration and possibility of the dental implant survival from a healthy distribution of the tension during the masticatory cycle. Numerous papers have recently recreated the masticatory system by engineering tools for simulating the long term stress over the dental, bone of the jaws, and prosthodontics components. FEM is a computer method for stress analysis. The effect of loading strengths over the dental implant elements and peri-implant bone can be recorded by applying the equivalent Von Mises stress, expressed in MPa. The difference in tension distribution is usually presented by different colors, where red is the maximum stress [2,4,7,16,17,18,19,20,21]. The present study was aimed at evaluating three different attachment systems for dental implants overdentures, to propose a better prosthodontics solution related to edentulous mandibular ridge restoration. 

The homogeneous distribution of tensional forces developed on dental devices during the masticatory cycles is influenced not only by the number and the position of the dental implants, but by the structural material, the shape, and the diameter of the singular component(s)’ geometry [1,2]. The present investigation was performed on different prosthetic elements of retention to point out possible failures related to any fracture of the structural components or any overload on the bone tissue. FEM was used to better evaluate the mechanical features of each implant-prosthetic component. The Ansys^®^ program was used to conduct the analysis, using three different implant systems:

(1) UNIVERSAL **ABUTMENT**,
(2) LOCATOR^®^ **ABUTMENT**,
(3) EQUATOR^®^ **ABUTMENT**.

**Figure 1:**
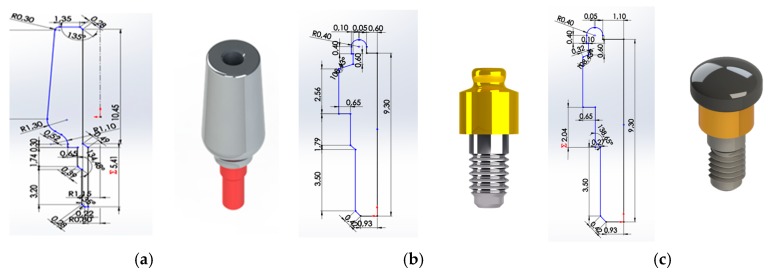
2D Sketch and rendering, (**a**) universal abutment, (**b**) locator abutment, (**c**) equator abutment, measured in mm.

A comparative analysis was performed to complete the systems, using a type of implant and various “pillars” and “abutments” connected by metric threading (Figure 1). The dimensions of the system components were not provided; therefore, it was necessary to go through a “reverse engineering” process. It was necessary to refer to prosthesis catalogues to acquire the initial measures. Furthermore, due to the lack of many measures, photos were used to derive them. The reverse engineering operation inevitably introduced approximations. Moreover, we used the obtained dimensions to create the geometry of the three-dimensional models in the SolidWork^®^ program. High importance was given to the materials (titanium alloy and bone) and to the parameters of the simulations, such as the definition of the contact surfaces, the mesh, and the loading conditions and the constraints. The results of the tests were available in the form of graphic simulations and data, which were compared to understand the optimal configuration between the systems analyzed. Finally, the Von Mises stress solutions were used and applied to the data.

## 2. Materials and Methods

Key parameters, which influence the accuracy of the results of the FEM system, were underlined. Among these, we considered, the detailed geometry of the system and the surrounding bone to be modeled, the boundary conditions and constraints, the material properties, the load conditions—repeated based on times related to the masticatory cycle, the bone—implant interface, the test of convergence, and the validation of the model.

Solid models of jaw arches, dental implants, and prosthetic overdenture elements were recreated from Roster images, which were processed using a 3D CAD “version 2014” in FEM. The analysis process was then divided into the following two phases as done in the pre-processing: the finite element model construction phase, and the post-processing: processing and representation of solutions [3,4].

### 2.1. Reverse Engineering

**Figure 2:**
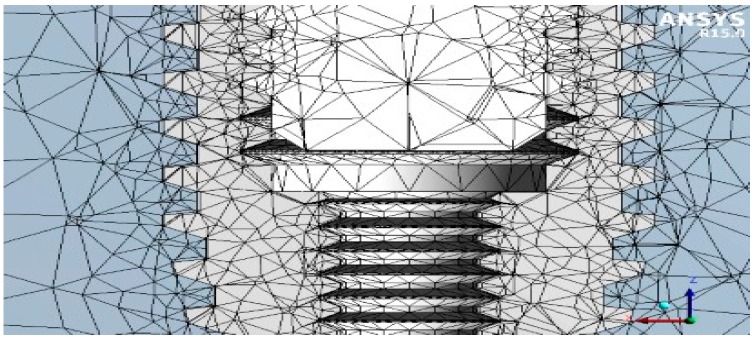
Reverse engineering.

The model dimensions were realized from the implant-prosthetic components and the images were made real using the small details of their physical-chemical characteristics, provided by the scientific literature and from the brand catalogues (Figure 2). The missing measurements were acquired using an electronic microscope, where the characteristics are reported in Table 1.

**Figure 3:**
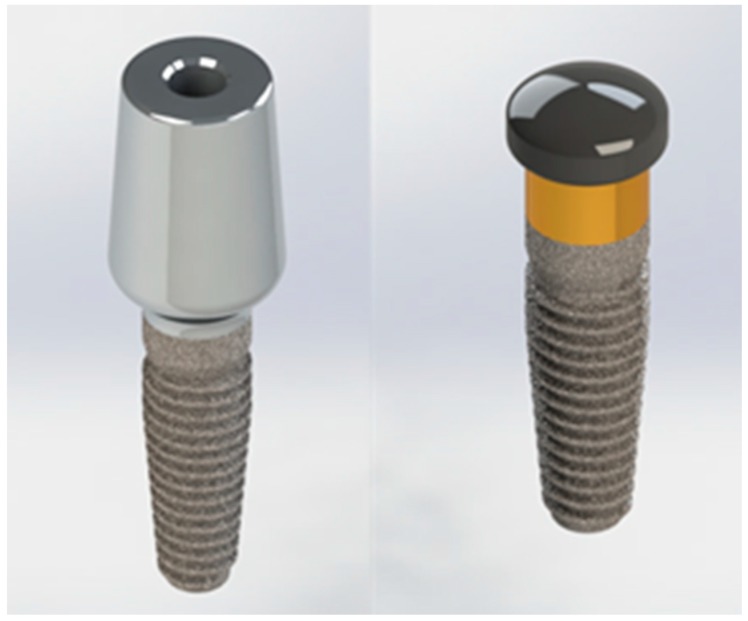
3D modelling.

The modeling phase was performed using SolidWork^®^, where the information was passed from the physical system to a mathematical model, extrapolated from the same number of variables and “filtering out” the remaining ones. Figure 3 shows an example of the reverse engineering process. 

### 2.2. Finite Element Analysis

**Figure 4:**
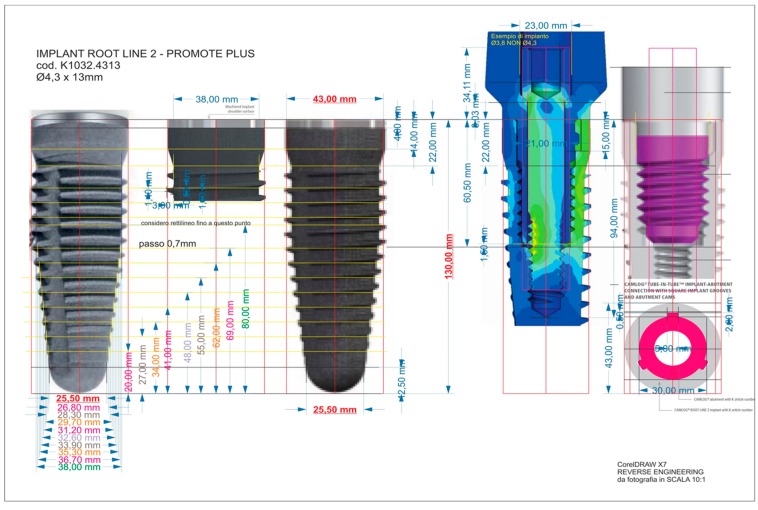
Reverse engineering of the dental implant used as anchorage.

Consequently, after obtaining these three-dimensional CAD models, the FEA jaw-implant-prosthesis was performed using the Ansys^®^ Workbench (Figure 4). A 3D linear static structural simulation was performed showing the relation (stress and strain) between the bone and the implant prosthodontics elements: fixture, Universal abutment, Equator^®^, and Locator^®^ systems.

#### 2.2.1. Mechanical Characteristic of the Materials

The same stress was applied to the different implants and the consequent strength distribution was evaluated. The properties of the materials were specified in terms of Young’s modulus, Poisson’s ratio, and density. The different physical behavior of the materials was considered, with respect to the occlusal loading and the lateral forces. The titanium alloy (Ti6Al4V) under examination could be considered homogeneous, linear, and isotropic, whilst the bone tissues (cortical and cancellous) that should be anisotropic were considered as orthotropic (Table 2). Therefore, different deformation features along the three main space directions in response to stress were noticed [1,3,4,5,8,21,22,23,24]. 

#### 2.2.2. Mesh

A method of discretization that foresees the use of a tetrahedral element with an independent algorithm and a lower limit of 0.2 mm in size was assigned to the whole elements of geometry. The 3D Hexa mesh was composed of many elements of second order SOLID186 (3D elements with 6 faces, 20 nodes, where each node had 3 degrees of freedom Dx, Dy, Dz). 

**Figure 5:**
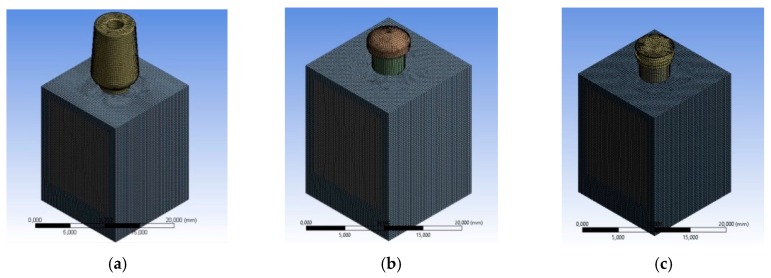
Meshing process, (**a**) mesh of the universal prosthesis, (**b**) mesh of the locator prosthesis, (**c**) mesh of the equator prosthesis.

**Figure 6:**
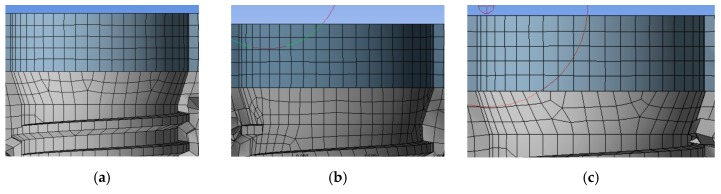
Meshing process, (**a**) mesh of the universal prosthesis, (**b**) mesh of the locator prosthesis, (**c**) mesh of the equator prosthesis.

Figure 5 shows the mesh of the three different implants and Figure 6 the shows node and element numbers for each implant. The number of tetrahedral elements was close to 230.000; while ensuring the lightness of the simulation, the number of elements testified to the accuracy of the model.

In a detailed analysis, it was noted that the mesh of the cortical bone was of a blue color, while the cancellous bone was gray in color (Table 3).

#### 2.2.3. Boundary Conditions

The components of the dental implants were tested using a compression load of 800 N [4,5,6,7,8]. All the loads were distributed on the prosthodontics components surface in contact (screwed) with the dental implant. The bone-implant and the bone—bone contact conditions established in this FEM analysis are reported in Table 4 as follows:

For all the threaded connections, a bolt pretension in accordance with the installation requirements was considered.

M [N cm] = K D P

K is a global coefficient that takes into account the friction coefficients on the thread and on the support surfaces, the screw coefficients: diameter/pitch ratio (and thus screw angle) in our case was worth 0.2.; D is the nominal thread diameter in mm; P is the preload or axial pre-tensioning of Newton that we intended to dare to the screw.

**Figure 7:**
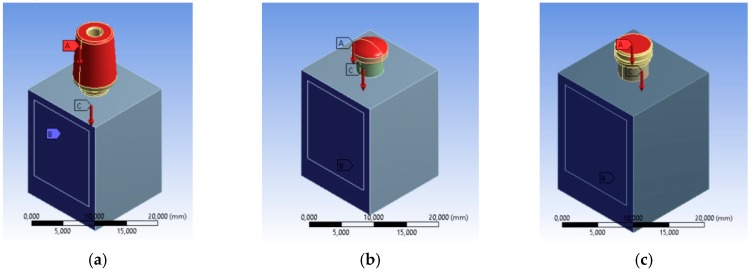
Loading conditions, constraint conditions and contacts, (**a**) universal prosthesis, (**b**) locator prosthesis, (**c**) equator prosthesis.

Specificaly:

1. implant/bone;
  P [N] = M/(0.2 D) = 40 N;
2. lacator abutment;
  P [N] = M/(0.2 D) = 50 N;
3. equator abutment;
  P [N] = M/(0.2 D) = 50 N;

With regards to the constraints, the lateral sides of the bone and the lower face were bound (Figure 7).

## 3. Results and Discussion

Compared to all the papers currently available in the scientific literature, this paper was the only study presenting a simulation that was as complete as possible, that is, contact between the surfaces of non-penetration with the friction and preloading of the connecting screw. In the past, to achieve a simpler and faster simulation, it was preferable to use a “joint” connection between the parties and not to take preload into account. Nevertheless, this was to the detriment of the truthfulness of the results. Instead, the authors found a good compromise to achieve results that were as close to reality as possible [3,4,5,6,7,8,9,24].

A CAD model of each component was recreated and then united in a single model with a relative constrain. At the same time, the aim of the research was to analyze the total stress on the three different geometries. A compression vertical load of 800 N was applied to the model. The Von Mises analysis was applied to the study to record the weak points of the system and around the bone tissue by color (red and yellow represented high stress).

A scale of values from 0 to 550 MPa was created to evaluate the stress in order to standardize the scale of values for all the simulations.

**Figure 8:**
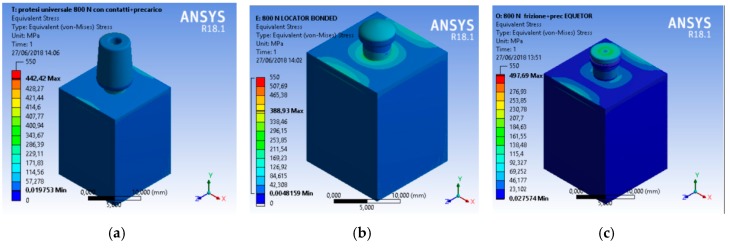
Von Mises results of the complex bone fixture and prosthodontics attachments. Three-dimensional view of the stress distribution, (**a**) universal prosthesis abutment, (**b**) locator prosthesis abutment, (**c**) equator prosthesis abutment (Figure 9, Figure 10 and Figure 11).

From an initial analysis, it was deduced that the whole system was not prejudiced (Figure 8). As observed, no system reached failure due to static rupture. In general, from the extension of the stressed areas, the following results were registered, that is, the system that stressed the bone less was the universal prosthesis, the system that most stressed the bone was the locator prosthesis, and the system that had the highest peak stress value was the equator prosthesis.

**Figure 12:**
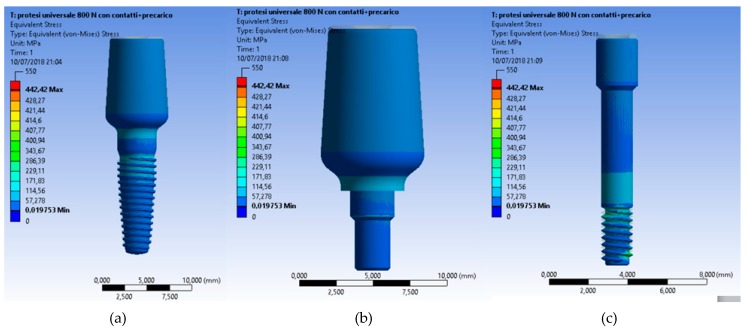
Von Mises results at (**a**) the universal abutment system prosthesis, (**b**) a particular of the abutment, (**c**) and the stress of the connection screw.

**Figure 13:**
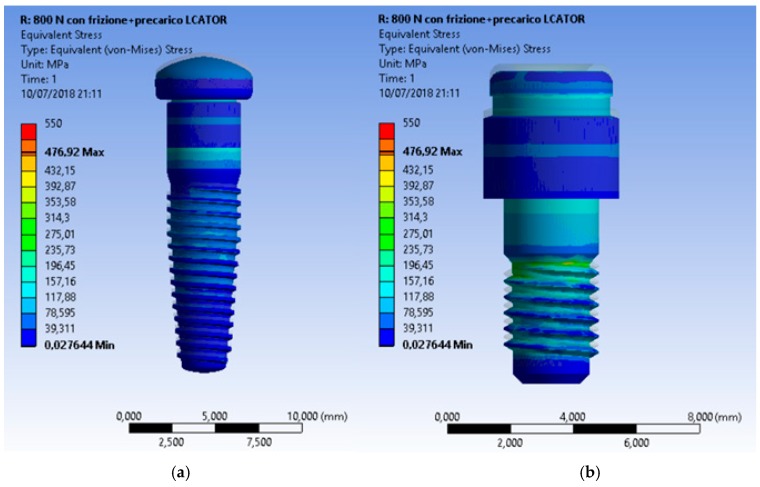
Von Mises results of the Locator^®^ system and a particular of the locator with no fixture.

**Figure 14:**
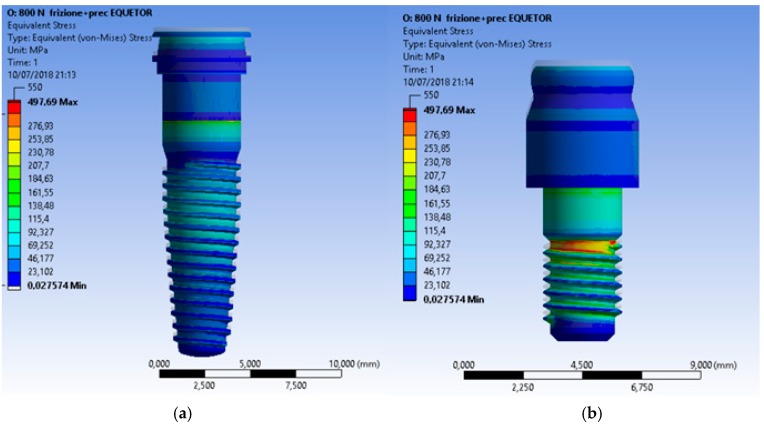
Von Mises results of the Equator^®^ system and a particular of the locator with no fixture.

In detail, in the thread area, the stresses reached were:

From the point of view of the dental fixture, it could be observed that: 

Even though all the recreated prosthodontics components represented a unique system involved in the masticatory cycle, the most stressed element of the fixture remained the connecting screw (Figure 12, Figure 13 and Figure 14).

In the second sample, the dental implant and abutment were investigated. The universal abutment had the most stress compared to the other cases. There was also an increase in the stress on the system compared to the previous case.

Finally, the last test showed how the most stressed system was the universal abutment. Therefore, the most recommended element is the geometry and shape of the universal abutment. However, there was less stress on the implant compared to the previous case.

For mandibular implant-based overdentures, different retention systems have been developed to fix the prosthesis over the dental implants. International literature agrees with regards to the minimum number of two dental implants located in the inter-foramina area [16,17,18,19]. However, regarding the retention systems, the topic remains quite debated [1,5,9,19]. The retention and stability characteristics were mainly provided by implants through attachments. Therefore, different attachment systems were created for connecting implant-retained mandibular overdentures to the underlying implants. Independent connections to each implant abutment with O-rings or splinting of implants with bar/clip attachments are the most common approaches that have been used. The bar overdenture is a popular choice because of its load sharing, but its cost is high and patients sometimes prefer to have higher stability with a lower cost [20,21,22,23].

Recently, several published papers underlined how in the field of implant dentistry, the knowledge of key parameters related to the bone implant integration phenomena still remained of significance for clinical long-term success. A deep investigation of the biomechanics of the oral cavity anatomy and physiology mechanism resulted in fundamental knowledge of the bone mechanical properties, as well as an accurate definition of the jawbone geometry [24,25,26,27,28,29,30,31,32].

Over the last 20 years, biomaterial shape and design have widely benefited from the integration of finite element analyses in the product development process. This system of analysis adopts an approach of computing reactions over a discrete number of points across the domain of interest. For medical device shape, this typically translates into verifying device performance in a virtual domain that is representative of its planned real-life application [24,25,26,27,28].

The advantages of FEM in the biomedical field are numerous. The most impressive advantage is related to the possibility that FEM can enable early device performance testing prior to costly prototyping and bench testing. Correspondingly, integration of the FEM process into medical device realization can decrease costs over the product development cycle. Such savings come to fruition by way of tentatively speeding up the process and reducing bench-testing iterations. 

From the other side, the disadvantages of FEM for medical device design reside mainly within the high expertise required to properly navigate the computational platform while avoiding making costly mistakes from ambitious misinterpretations [18,30,31,32].

Therefore, even though the method was able to create all the micromechanical characteristics of the medical device, it still remains hard to reproduce all the body clinical features placed into a dynamic contest. The data of the presented investigation offered a challenge compared to the recent literature. The presence of a K coefficient for avoiding a boundary system could be classified as a new step method for considering the integration between a static medical device and a dynamic human body wears. 

Specifically, in the field of dentistry, the geometry of several prosthetic devices for retained overdenture structure is widely treated in recent literature for evaluating the integration and the wear related to the masticatory cycles. The Locator^®^ system (Zest Anchor, Escondido, CA, USA) has been widely investigated, with several published documents using in vitro and clinical study. Its mechanical features are related to its small shape size, its retention capacity over a long time, and its wide tolerance to being used with high angulation dental implants. The Equator^®^ system (Rhein 83, Bologna, Italy) has been recently studied because it was commercially launched in 2007. This attachment can be used for both the overdenture with direct connection and for the overdenture to connect a secondary structure. As in the present investigation, the OT Equator showed similar retention capacity to the Locator system [31,32,33,34,35,36,37,38].

A review and meta-analysis on dental implant overdenture attachments and their influence on peri-implant bone loss performed by Keshk et al. and published in 2017, revealed how there are no statistically significant differences between the type of overdenture attachment analyzed with regard to marginal bone loss, bleeding index, gingival index, and plaque index. In conclusion, no significant differences in prosthodontics maintenance and peri-implant conditions could be related to a different overdenture retained attachment system. This result was also highlighted in the present study; however, the shape of the two systems were characterized by different geometry, and thus, could be reflected through different but not significant strength distributions during the masticatory cycle [39].

## 4. Conclusions

The data from the present investigation clearly underlined how the locator and equator system offered better stress distribution compared to the traditional universal abutment.

Moreover, within the limitation of the present “in vitro” study, the Von Mises analysis underlined how both prosthodontics overdenture retainer systems tolerated well the masticatory stress, though the Equator^®^ system involve less of the bone peri-implant tissues. Specifically, the results could be interpreted as follows:

The Equator^®^ and Locator^®^ retention systems offered adequate retention systems and overdenture prosthesis support. The universal abutment supported low stress up until about 442 MPa. Therefore, this in vitro study underlined how the shape of the Locator^®^ distributes the stress over the dental implant and that the gum retainer could be supported for a long time as compared to the other systems. The limit of the components fracture occurred at 476.92 MPa. Moreover, the shape of the Equator^®^ retained system seems to collect the strength over the head of the retainer. These conditions favored the higher stress on the retainer gum. The advantage was related to the minor stress located around the peri-implant bone tissue and fixture. Moreover, the Locator System can overload and support stress until 497.69 MPa.

## Figures and Tables

**Figure 9:**
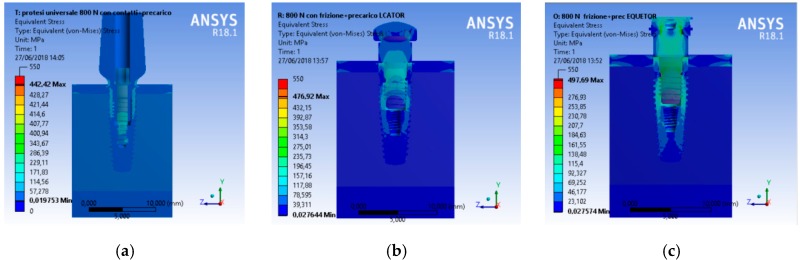
Von Mises results of the complex bone fixture and prosthodontics attachments, (**a**) universal prosthesis, (**b**) locator prosthesis, (**c**) equator prosthesis.

**Figure 10:**
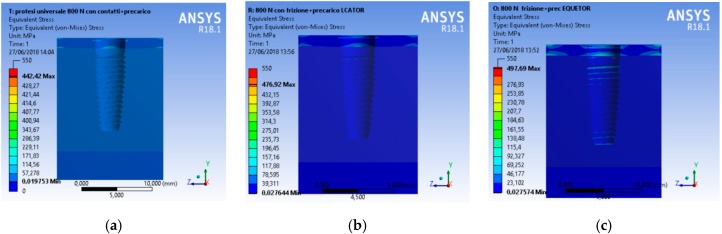
Von Mises results referred to the bone at the maximum stress, (**a**) universal prosthesis, (**b**) locator prosthesis, (**c**) equator prosthesis.

**Figure 11:**
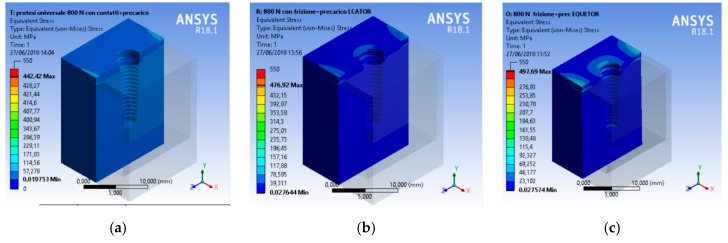
Von Mises results, (**a**) universal prosthesis, (**b**) locator prosthesis, (**c**) equator prosthesis.

**Table 1:** Electronic microscope properties.

**Resolution**	640 × 480 pixel
**Zoom**	5×
**Color**	Black
**Software**	Windows 2000/2003/XP/Vista/Linux/10
**Dsp**	24 bit
**Software bit**	Usb 2.0–Usb 1.1
**Model**	Usb

**Table 2:** Properties of the materials.

Material C	Cortical Bone	Cancellous Bone	Ti6Al4V
Density	1.8 g/cm^3^	1.2 g/cm^3^	4.510 g/cm^3^
E**_xx_**	9.60 GPa	0.144 GPa	105 GPa
E**_yy_**	9.60 GPa	0.099 GPa	105 GPa
E**_zz_**	17.8 GPa	0.344 GPa	105 GPa
**ν_xy_**	0.55	0.23	0.37
**ν_yz_**	0.30	0.11	0.37
**ν_xz_**	0.30	0.13	0.37
G**_xy_**	3.10 GPa	0.053 GPa	38.32 GPa
G**_yz_**	3.51 GPa	0.063 GPa	38.32 GPa
G**_xz_**	3.51 GPa	0.045 GPa	38.32 GPa

**Table 3:** Number of elements and nodes, respectively, of the three analyzes.

	UNIVERSAL	LOCATOR	EQUATOR
Nodes	902,969	878,286	899,799
Elements	234,022	230,457	236,527

**Table 4:** Frictional value considered for each analyzed system. The processing considered K as a global coefficient applied to the thread of the dental implant’s supported surface.

**Equator Abutment**			
**Target Bodies**	**Contact Bodies**	**BONDED**	**FRECTIONAL**
external retention matrix	inner sheath	//	
inner sheath	abutment	//	
implant	abutment		0.3 K
implant	cortical bone		0.2 K
cortical bone	cancellous bone	//	
implant	cancellous bone		0.2 K
**Locator Abutment**			
**Target Bodies**	**Contact Bodies**	**BONDED**	**FRECTIONAL**
retention insert	abutment	//	
implant	abutment		0.3 K
implant	cortical bone		0.2 K
cortical bone	cancellous bone	//	
implant	cancellous bone		0.2 K
**Universal Abutment**			
**Target Bodies**	**Contact Bodies**	**BONDED**	**FRECTIONAL**
abutment	screw		0.3 K
implant	screw		0.3 K
implant	abutment		0.3 K
implant	cortical bone		0.2 K
cortical bone	cancellous bone	//	
implant	cancellous bone		0.2 K
